# Measurement of SUVs-Maximum for Normal Region Using VOI in PET/MRI and PET/CT

**DOI:** 10.1155/2014/194925

**Published:** 2014-02-04

**Authors:** Jeong Kyu Park, Sung Kyu Kim, Ihn Ho Cho, Eun Jung Kong

**Affiliations:** ^1^Department of Radiologic Technology, Daegu Health College, Youngsong-ro 15, Buk-gu, Daegu 702-722, Republic of Korea; ^2^Department of Therapeutic Radiology & Oncology, College of Medicine, Yeungnam University, Nam-gu, Daegu 705-717, Republic of Korea; ^3^Department of Nuclear Medicine, College of Medicine, Yeungnam University, Nam-gu, Daegu 705-717, Republic of Korea

## Abstract

The purpose of this research is to establish an overall data set associated with the VOI (Volume of Interest), which is available for simultaneous assessment of PET/MRI and PET/CT regardless of the use of contrast media. The participants as objects of this investigation are 26 healthy examinees in Korea, SUV (standardized-uptake-value)s-maximum evaluation for whole-body F-18 FDG (fluorodeoxyglucose) PET/MRI image using VOI of normal region has exhibited very significant difference to that for whole-body F-18 FDG PET/CT image (significant probability value (*P*) < 0.0001). However, there appeared high correlation between them in view of statistics (*R*-square (*R*) > 0.8). It is shown that one needs to decide SUVs-maximum for PET/MRI with the reduction of 25.0~26.4% from their evaluated value and needs to decide with the reduction of 28.8~29.4% in the same situation but with the use of contrast media. The use of SUV_LBM_-maximum (SUV_Lean Body Mass_-maximum) is very advantageous in reading overall image of PET/CT and PET/MRI to medical doctors and researchers, if we consider its convenience and efficiency. We expect that this research enhances the level of the early stage accurate diagnosis with whole-body images of PET/MRI and PET/CT.

## 1. Introduction

The introduction of recently developed imaging apparatus PET/MRI that combines PET (positron emission tomography) and MRI (magnetic resonance imaging) gives very promising anticipation as a step for new and more powerful acquisition of body images [1]. The wide range merit of MRI, compared to CT (computed tomography), includes the enhancement of contrast and diffusion, its availability of perfusion, improved functional image for bone marrow positive region accomplishable from MRI spectroscopy, and high resolution for detecting tumors in, for example, soft tissue and liver, in addition to substantial reduction of the risk of radiation exposure to the patient [2–8].

The merit of the one-unit PET/CT is that not only can we establish CT data using X-ray but also it enables us to achieve attenuation correction (AC) for PET image using CT value (Hounsfield unit, HU) of CT image [4]. However, there is no direct relevance between MRI signal and the attenuation of radiology and it is known to be impossible to use existing MRI data in performing AC for PET data [5]. Accordingly, a new method for analyzing the image data obtained from PET/MRI is required and the necessity for using PET data merged to MRI data has recently emerged in whole-body images treated with AC. In particular, a method (Biograph mMRI, Siemens Medical Solutions) of AC for recently introduced hybrid whole-body PET/MRI is developed on the basis of 2-point Dixon-based MRI pulse sequence [6].

Standardized uptake values (SUVs) are an important index for evaluating the accuracy of cancer diagnosis carried out via inspection of F-18 FDG (fluorodeoxyglucose) PET/CT and the effects of their cure. Hence, if we consider that F-18 FDG sticks fast to some parts of the internal organs physiologically, the pathological data can be estimated from SUVs. For the case of the intake of F-18 FDG, the diversity of SUVs is essential in order to understand normal organization of the human body [7].

Many researchers have evaluated the equation of SUVs associated with biological or physical factors after setting up the size of the region of interest (ROI) [8]. Because their average and maximum values are different depending on ROI, it is necessary to establish ROI where maximum absorption is exhibited in order to evaluate the degree of malignancy of cancers [9].

Recently, many researchers take body weight (BW) considering the weight of patient, body surface area (BSA) considering the weight without fat, and lean body mass (LBM) considering the area of the surface of body as reference factors for the results they obtained [10, 11] and it is reported that the method of measuring SUV using each patient's LBM gives the most exact results [11]. In particular, if we consider that unified PET/MRI equipment, which is the most advanced medical imaging devices, is very recently started to be installed in hospitals, it is not easy to acquire (or secure) the SUVs data for PET/MRI. Although the information associated with 3-dimensional volume in the measurement of SUVs offers more precise data than others, any results of research performed using VOI for PET/MRI data are not yet reported in the medical society as far as we know. Therefore, the research on SUVs-maximum using 3-dimensional VOI is highly required, even if there is a method for obtaining SUV_LBM_-maximum using ROI relevant to 2-dimensional area in PET/CT.

In this work, we intend to enhance the level of early/precise diagnosis for positive regions such as cancers and inflammations via the research of SUVs-maximum using 3-dimensional VOI, which is recently enabled with the introduction of PET/MRI equipment in hospitals. In particular, we try to establish the data sets which enable us to simultaneously evaluate images of PET/CT and PET/MRI regardless of the use of contrast media. The final purpose of this research is to compare and analyze SUVs-maximum statistically for whole-body image obtained from PET/CT and PET/MRI for healthy examinees.

## 2. Materials and Methods

### 2.1. Subject of Examination

The number of patients who became the object of our survey is 26. They had taken PET/MRI examination immediately after the examination of whole-body FDG PET/CT from July 2012, to August 2012. This research is carried out under the permission of Institutional Review Board (IRB) of Korea. They are six men and twenty women. Their age is distributed from 35 to 75 and the average age is 52.5. Distributed stature is from 145 cm to 177 cm and their average is 160.0 cm. The range of weight is from 40 kg to 76 kg and their average weight is 57.8 kg. The number of examinees who take contrast media is 16 and who do not take is 10.

### 2.2. Methods

#### 2.2.1. The Acquisition of PET/CT Images

The examinees fasted at least six hours before they take examination and F-18 FDG is injected into the necessary parts of their body after meeting a required condition that their checked blood-glucose level is lower than 180 mg/dL. F-18 FDG 8.14 MBq/kg is injected in the examinees' vein and, after 60~90 minutes, the PET/CT (Discovery VCT, GE Milwaukee, USA) images are acquired at first. The AC for the obtained CT image (thickness: 3.75 mm; 140 kVp; 120 mA) is fulfilled and then we obtained 7~9 beds of 3D-mode emission images in the same range as the primary CT scan. The time taken for each scan is about 3 minutes. The PET image is reconstructed to be OSEM (ordered-subsets expectation maximization method) algorithm mode, and AC is carried out using CT image.

#### 2.2.2. The Acquisition of PET/MRI Images

The examinees who had taken PET/CT examination had been immediately examined PET/MRI (Biograph mMRI, 3T Siemens, Erlangen, Germany) in order to get the required images. After the first image from head to proximal femur is obtained (it takes about 19 seconds), 4 beds of 3D mode emission image are obtained in the same range as the CT scan, where the acquirement of each bed takes two minutes. PET images are reconstructed to be the OSEM algorithm mode and AC is carried out using Dixon VIBE MRI images.

Four body coils are set up in front of head coil in order to optimize signal-to-noise ratio (SNR) and spine clusters are equipped in the rear of it. Coronal plane T_1_ weight MRI pulse sequence based on 2-point Dixon 3D volume is applied under the condition of inducting regular breathing and, after that, the scanning table is moved to scan along head/neck, breast, abdomen, and pelvis in turn.

The variables relevant to obtaining MRI image are as follows: integrated parallel acquisition technique factor 2, voxel size 4.1 × 2.6 × 3.1 mm^3^ (in-plane resolution × slice thickness), acquisition time 18 s, repetition time (TR)/echo time (TE) 3.6/1.225, matrix 79 × 192, number of excitations 1, FOV 500 mm, phase FOV 72%, 1 slab with 128 slices, slice thickness 2.6 mm, flip angle 10°, and bandwidth 960 kHz.

To minimize artifacts produced through irregular breathing, *k*-space acquisition in the middle is selected. Contrast media are not used in the inspection taken with MRI and raw images that automatically generate T_1_ weight in-/out-of-phase, water-only image, and fat-only image are used.

#### 2.2.3. Process for the Acquirement of Data

PET data has been attenuation corrected and they are reconstructed as a mode of OSEM data. Attenuation maps are obtained from CT data produced by two lines in the existing mode which uses postprocess software tool of PET/CT scanner.

Since inaccurate registration induces a significant problem in determining proper quantity of SUVs, attenuation correction factors are chosen delicately in this research and they are 0.1/cm for background, 0.018/cm for lung, 0.086/cm for fat, and 0.1/cm for soft tissue. This AC is used for reconstructing PET image on the basis of regularization of simulated MRI-based AC (PETAC_MRI) for overall items such as in the case of PETAC_CT.

## 3. Analysis

### 3.1. Analysis of Images

Raw data, CT data, PETAC_CT, and PETAC_MRI associated with the four parts of image (T emphasis in-/out-of-phase, fat-only, and water-only) produced on the basis of Dixon MRI pulse sequence are transferred to workstation (Syngovia-3D Fusion, Siemens Medical Solution, Erlangen, Germany). Although all images enrolled in syngo.via are automatically evaluated, they can also be locally adjusted by hand if necessary. In PETAC_MRI, all uptaken portions are exactly represented in the merged anatomic image. Coronal plane, sagittal plane, and transverse plane are represented in monitor using 3D Fusion MM Oncology in order to evaluate the ability of quantity representation of PET/CT and PET/MRI. We referred [12] for anatomical position of VOI in the image of transverse plane and VOI is selected with the aid of medical doctors.

**Figure 1:**
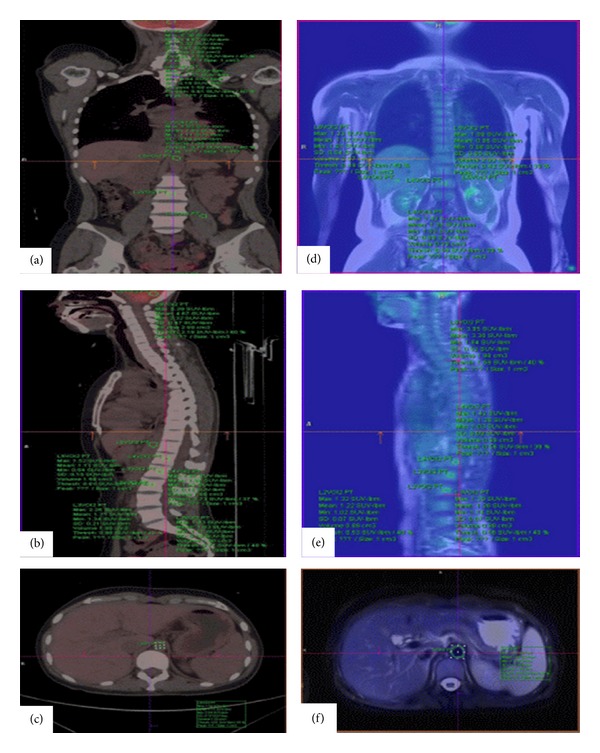
(a)~(c) SUVs measurements confirm anatomical location in the PET/CT coronal, sagittal, and transverse images; (d)~(f) SUVs measurements confirm anatomical location in the PET/MRI coronal, sagittal, and transverse images.

Liver (segment 6, 3 cm^3^), spleen (splenic hilum, 2 cm^3^), aorta (pancreas, 1.5 cm^3^), bone marrow (lumbar 1, 2, and 3; body center, 1 cm^3^), lumbar 5 (from upper plate to Multifidus, 1 cm^3^), and cerebellum (center, 2 cm^3^) are selected and drawn. The errors of the range of measurements are confined within ±0.01% in this drawing. We also fitted anatomical positions of volume in coronal plane, sagittal plane, and transverse plane using the two lines (Figure 1).

SUVs are evaluated using maximum value of LBM, BW, and BSA after drawing VOI within normal tissue. Although brain is, in general, excluded in measurements of SUVs due to the effects of cortical bone and the use of contrast media accompanies latent danger [13], we in this research not only included cerebellum but also used contrast media in PET/CT images in order to secure the universality of the results. Although peak value is recommended as PET response criteria in solid tumors (PERCIST), it is not observed in normal tissue [12].

If we set a volume corresponding to a region that is needed to be measured, SUVs are measured automatically and they are evaluated by an average of 40% isocontour VOI. When measuring PET/MRI images together with PET/CT for the same patient, we have identified whether the variables of SUVs such as stature and weight are corresponding or not.

### 3.2. Statistical Analysis

We used SPSS software (SPSS Inc., Version 20.0) designed for Microsoft Windows operating system in managing all statistical processes with measured data. Linear regression analysis for SUVs-maximum of PET/CT and PET/MRI is performed and corresponding comparison of each normal area is carried out according to corresponding sample *t*-test. We regarded that *P*-value which is the significant probability in statistics is significant only when it is lower than 0.05 in all statistical analyses.

## 4. Results

### 4.1. Comparison of SUVs-Maximum for PET/CT and PET/MRI without Using Contrast Media: Comparison of SUV_LBM_-Maximum for PET/CT and PET/MRI without Using Contrast Media

The results of linear regression analysis performed via computing variation of SUV_LBM_-maximum for patients taking both PET/CT and PET/MRI shows very significant correlation between PET/CT SUV_LBM_-maximum and PET/MRI SUV_LBM_-maximum, which is 0.873 (*P* < 0.01). Equation of regression derived from this analysis is as follows:

(1) $$\begin{array}{c} Y=-0.495+0.974x. \end{array}$$

The meaning of 95% confidence interval for regression coefficient *B* is that PET/MRI SUV_LBM_-maximum increases to the range 0.48–1.46 when PET/CT SUV_LBM_-maximum grows 1 unit (Table 1).

### 4.2. Comparison of SUV_BW_-Maximum for PET/CT and PET/MRI without Using Contrast Media

The linear regression analysis performed via variation calculation of SUV_BW_-maximum for patients taking both PET/CT and PET/MRI resulted in the correlation between PET/CT SUV_BW_-maximum and PET/MRI SUV_BW_-maximum is 0.924 which being very significant (*P* < 0.0001). The regression equation derived from this analysis is

(2) $$\begin{array}{c} Y=-1.339+1.221x. \end{array}$$

The meaning of 95% confidence interval for regression coefficient *B* is that PET/MRI SUV_BW_-maximum increases the amount of the range 0.770–1.672 as the PET/CT SUV_BW_-maximum grows 1 unit (Table 2).

### 4.3. Comparison of SUV_BSA_-Maximum for PET/CT and PET/MRI without Using Contrast Media

The results of linear regression analysis obtained from variational calculation of SUV_BSA_-maximum for patients taking both PET/CT and PET/MRI exhibit that the correlation between PET/CT SUV_BSA_-maximum and PET/MRI SUV_BSA_-maximum is 0.661, which is somewhat significant (*P* < 0.05). The regression equation derived from this analysis is as follows:

(3) $$\begin{array}{c} Y=-0.018+0.762x. \end{array}$$

The meaning of 95% confidence interval for regression coefficient *B* is that PET/MRI SUV_BSA_-maximum increases the amount of the range 0.011–1.535 as the PET/CT SUV_BSA_-maximum grows 1 unit (Table 3).

### 4.4. Comparison of SUVs-Maximum for PET/CT and PET/MRI Using Contrast Media: Comparison of SUV_LBM_-Maximum for PET/CT and PET/MRI Using Contrast Media

The analysis of linear regression using variational calculation of SUV_LBM_-maximum for patients taking both PET/CT and PET/MRI shows that the correlation between PET/CT SUV_LBM_-maximum and PET/MRI SUV_LBM_-maximum is 0.748 which is very significant. The regression equation obtained from this analysis is

(4) $$\begin{array}{c} Y=0.192+0.613x. \end{array}$$

The meaning of 95% confidence interval for regression coefficient is that PET/MRI SUV_LBM_-maximum increases the amount of the range 0.27–0.95 when PET/CT SUV_LBM_-maximum grows 1 unit (Table 4). It turned out that the case without using contrast media exhibits higher correlation as well as broader range of confidence interval than those for the case using contrast media.

### 4.5. Comparison of SUV_BW_-Maximum for PET/CT and PET/MRI Using Contrast Media

The results of linear regression analysis obtained from the variational calculation for the case of the patients taking both PET/CT and PET/MRI reveal that the correlation between PET/CT SUV_BW_-maximum and PET/MRI SUV_BW_-maximum is 0.749, which is somewhat significant (*P* < 0.01). The regression equation obtained from this analysis can be written as

(5) $$\begin{array}{c} Y=0.545+0.508x. \end{array}$$

The meaning of 95% confidence interval for regression coefficient is that PET/MRI SUV_BW_-maximum increases the amount of the range 0.225–0.790 when PET/CT SUV_BW_-maximum grows 1 unit (Table 5).

### 4.6. Comparison of SUV_BSA_-Maximum for PET/CT and PET/MRI Using Contrast Media

From the results of variational calculation of SUV_BSA_-maximum for patients taking both PET/CT and PET/MRI, we conclude that the correlation between PET/CT SUV_BSA_-maximum and PET/MRI SUV_BSA_-maximum is 0.648 which is significant (*P* > 0.05). The equation of regression derived from this analysis is The meaning of 95% confidence interval for regression coefficient is that PET/MRI SUV_BSA_-maximum increases the amount of the range 0.569–1.048 as the PET/CT SUV_BSA_-maximum grows 1 unit (Table 6).

(6) $$\begin{array}{c} Y=-0.034+0.658x. \end{array}$$

### 4.7. *t*-Test of Corresponding Sample for Each Normal Region Using SUVs-Maximum: *t*-Test of Corresponding Sample for Each Normal Region Using SUV_LBM_-Maximum

All of SUV_LBM_-maximum in PET/MRI image decreased significantly with the reference PET/CT image for the eight normal areas taken without using contrast media (*P* < 0.05). It turned out that PET/MRI exhibits a total of 26.3% difference with the reference to PET/CT: spleen showed the largest difference (32.1%) whereas aorta the smallest difference (18.2%). All of SUV_LBM_-maximum is significantly decreased with the reference to that of the PET/CT image of the eight normal areas taken with the use of contrast media (*P* < 0.01).

A total of 29.3% difference appeared in PET/MRI with reference to PET/CT, the highest difference is appeared in spleen (35.8%), and the lowest difference is in aorta (22.8%). It turned out that the case using contrast media exhibits 3.0% higher difference than that without using contrast media (Table 7).

### 4.8. *t*-Test of Corresponding Sample for Each Normal Region Using SUV_BW_-Maximum

All of SUV_BW_-maximum in PET/MRI image appeared to decrease significantly with reference the eight areas of PET/CT image without using contrast media (*P* < 0.05). There appeared 25.0% difference in PET/MRI image with reference to PET/CT image and the largest difference is shown in lumbar 5 (33.6%) and the smallest difference in aorta (15.7%). All of SUV_BW_-maximum in PET/MRI image decreased significantly with reference to eight normal areas PET/CT image obtained with the use of contrast media (*P* < 0.01). PET/MRI image showed a total of 28.8% difference from that in PET/CT image: the largest difference appeared in liver (35.8%) whereas the smallest in aorta (17.6%). The difference of the case using contrast media is higher by 3.7% than that without using contrast media (Table 8).

### 4.9. *t*-Test of Corresponding Sample for Each Normal Region Using SUV_BSA_-Maximum

All of SUV_BSA_-maximum in PET/MRI image are decreased significantly compared to those of the eight normal areas of PET/CT image without using contrast media (*P* < 0.05). SUV_BSA_-maximum for PET/MRI showed a total of 26.4% difference from that for PET/CT: the largest difference appeared in lumbar 5 (33.3%) whereas the smallest in aorta (18.0%). All of SUV_BSA_-maximum in PET/MRI image are decreased significantly compared to those of the eight normal areas of PET/CT image obtained with the use of contrast media (*P* < 0.01). PET/MRI image shows a total of 29.4% difference from that of PET/CT image: the largest difference appeared in spleen (36.2%) whereas the smallest difference is in aorta (18%). The case using contrast media exhibits 2.9% higher difference than that without using contrast media (Table 9).

## 5. Discussion

**Figure 2:**
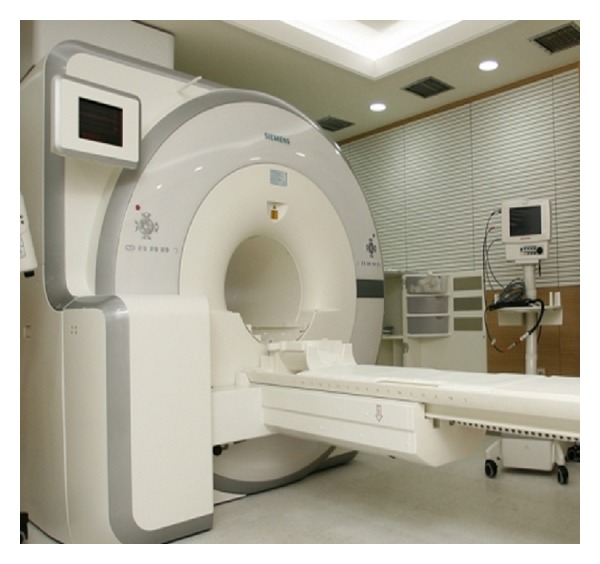
PET/MRI scanner (Biograph mMRI, 3T Siemens, Erlangen, Germany).

In this research, we evaluated and compared various statistical data relevant to SUVs-maximum for normal area, which are measured from the most up-to-date single-unit PET/MRI, with those for previously existing PET/CT. The PET/MRI equipment used here is the one that is firstly introduced in Korea (Figure 2).

Though AC for PET image in the PET/CT is fulfilled using HU offered from CT, a new approach for AC is necessary in the case of obtaining whole-body PET/MRI image and, according to this, Martinez-Möller et al. obtained PET/MRI image by representing anatomical positions in a similar way to that in CT using Dixon MRI pulse sequence within short time [6].

Most primitive trials which applied attenuation map on the basis of anatomy failed to give satisfactory results due to the diversity of patients [5]. After that, very short echo time technique which enables distinguishing air from cortical bone is suggested in the study of neurological image [14] and an automatic pattern recognition technique connected with anatomical positions is developed [15]. Automatic pattern recognition technique for whole-body image is initially suggested as an adoption of automatic sorting method for specimen of other tissues [16]. Martinez-Möller and coresearchers reported a technique including a sorting method of attenuation map for the four parts (background, lungs, fat, and soft tissue), on the basis of 2-point Dixon MRI pulse sequence. However, there is a known weak point in their method, which is that it neglects latent effects of cortical bone [6].

CT images carried out AC are necessary in representing anatomical correlation in PET/CT and, in many cases, they are useful in detailed description of, for example, progress of new living things, trouble in bones, lungs, and image analysis in lymphatic glands, although they do not replace existing CT images [17]. The range of scan for PET/MRI in anatomical view covers the whole body, which is identical with that for PET/CT. Actually, the examination of whole-body with CT needs 20~30 seconds and the acquisition of T weight image with FSE (fast spin echo) pulse sequence used exclusively for brain takes 3~4 minutes. Hence, the acquisition of whole-body image with MRI is restricted by time.

The resolution of PET/MRI images is very high provided that regular breathing of the patient is kept. Because the acquisition of PET image requires 2 or 3 minutes per bed, it is possible to obtain MRI image simultaneously, which requires just 19 seconds. This may enable saving time needed for inspection, leading to improving some efficiency providing convenience with low medical cost. In principle, for whole-body PET/MRI is highly expected for finding liver disease, head and neck, and disease in cranial cavity using the techniques offered by MRI [15] and the reason why these show pathologically good image than the CT data is that the contrast of soft tissue for that regions is high.

Data for Dixon-based MRI pulse sequence relevant to transferring of bones, frequently shown in the previous MRI largely used before the development of single-unit device, appears in T weight image. Fat-only image of Dixon pulse sequence is suitable for bone marrow partially composed of fat and moving with the induction of new cell [13]. The transition of the lymphatic gland appears well in T weight in-phase image [18]. Though anisotropic pulse sequence of previously existing MRI cannot exactly describe pathological and anatomical position, isotropic 3D pulse sequence admits to carry out various plans without difficulty.

There is a trend that space resolution of PET/MRI (4.1 × 2.6 × 2.6 mm^3^, 94 × 256 matrix) is more or less degraded when it is compared to that of CT (512 × 512 matrix and a soft tissue kernel). However, the research of Martinez-Möller shows that the enhancement of contrast for soft tissue [6], including the appearance of different image set, does not mean that there is a difference in that signal in morphological and functional point of view. There can be a difference of image interpretation between PET/MRI and PET/CT, that is, the possible inhomogeneities in four different organs that are neglected in MRI in contrast to the decay of radiology in CT.

We compared our research results with SUV_BW_-maximum results of another paper whose images are evaluated in normal area using 2-dimensional ROI [7]. The value of SUV_BW_-maximum is known from the research of Zincirkeser as follows: cerebellum 10.5, liver 5.0, spleen 4.1, aorta 3.1, and lumbar 1 5.2 for man and cerebellum 10.1, liver 3.8, spleen 3.2, aorta 2.9, and lumbar 1 4.5 for woman. However, in this research, there appeared cerebellum 6.5, liver 2.4, spleen 1.8, aorta 1.2, and lumbar 1 2.5 for man and cerebellum 7.4, liver 2.6, spleen 2.0, aorta 1.9, and lumbar 1 2.4 for woman. This means that SUV_BW_ for the result of this research using 3D VOI is low which brings up a necessity for clarify the data via measuring more SUVs in overall regions.

The research of Martinez-Möller shows that correlation coefficients of SUV_BW_-mean in the evaluation of cancer tissue with PET/CT and PET/MRI are 0.9975, 95% with confidence interval 0.9961~0.9948 (*P* < 0.0001) [19]. The result of our research of correlation coefficient of SUV_BW_-mean for normal area is 0.945, 95% with confidence interval 0.273~0.922 (*P* < 0.0001). From this difference, we conclude that cancer tissue accumulates more FDG than normal area.

Another difference in scintillator is that fluorescent materials used in PET/CT and PET/MRI are made in the same company and that is lutetium oxyorthosilicate fluorescent material. Concerning this research, LSO fluorescent material is used for PET/MRI and BGO (bismuth germanium oxide); fluorescent material is used for PET/CT, leading us to conjecture that there is a difference of sensitivity between them.

In this research, the cases in which correlation coefficient is higher than 0.8 are SUV_LBM_-maximum and SUV_BW_-maximum. In general statistics, we see that correlation coefficients higher than 0.8 are regarded as high correlation, between 0.6~0.8 are normal, 0.4~0.6 are low, and below 0.4 are evaluated as uncorrelated. Hence, when we evaluate image regardless of using contrast media, the use of SUV_LBM_-maximum is recommended. In fact, SUV_LBM_-maximum is also used in previous actual researches since the average value of standardized uptake values is unstable every time when we acquire them due to irregular size of positive regions [9].

SUVs are affected by various factors which are enumerated as follows. At first, there is a method in which physical constitution of patient is evaluated relatively by putting the specific gravity of body as 1. The index of blood glucose is a factor that disturbs the absorption of FDG in cancer. In case of PET, since the positive region reveals higher absorption rate than their environment, if the size of the measuring object is not sufficiently larger than the space resolution (at least more than 5 times), the measured radioactivity is lower than the actual one.

The data of SUVs-maximum measured from the normal eight positions are uniform for the case of F-18 FDG. However, in general, there is a limit when diagnosing cancer and inflammation in connection with high absorption rate because it is difficult to obtain physiological upper threshold. Hence it is required to know SUVs-maximum of normal area in PET/CT and PET/MRI as an appropriate counterplan to reduce false positivity and false negativity [7].

Considering conveniency and efficiency for medical doctors and researchers, it is useful to use SUV_LBM_-maximum when we read overall image of PET/CT and PET/MRI. We expect future-oriented active research for PET/MRI image in the upcoming studies.

## 6. Conclusions

SUV_LBM_-maximum evaluation using VOI of normal area for whole-body F-18 FDG PET/CT and whole-body F-18 FDG PET/MRI exhibits very significant difference with reference to PET/CT (*P* < 0.0001). In addition to this, they exhibit high correlation statistically (*R* > 0.8) and SUV_LBM_-measured shows the lowest rate of reduction among various data managed regardless of using contrast media. It is necessary to judge with reduction of 25.0~26.4% when we evaluate SUVs of PET/MRI, whereas we should reduce 28.8~29.4% when we judge it with the use of contrast media.

## Figures and Tables

**Figure 3:**
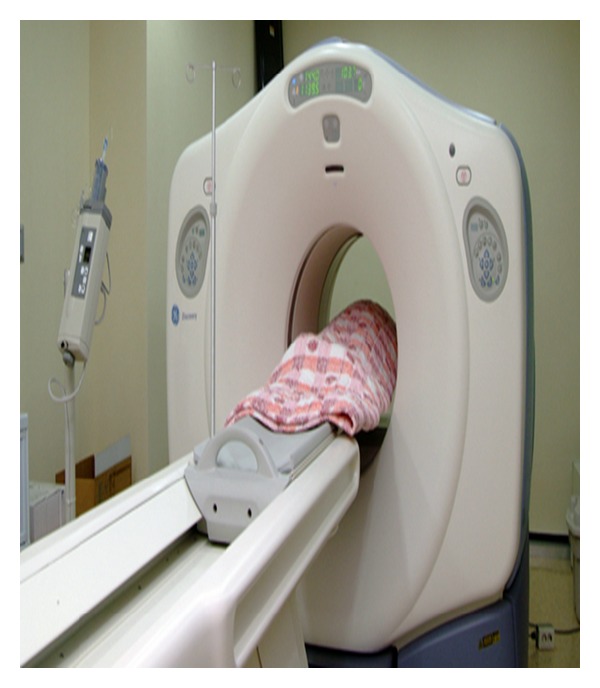
PET/CT scanner (Discovery VCT, GE Milwaukee, USA).

**Table 1:** Comparison of PET/CT SUV_LBM_-maximum and PET/MRI SUV_LBM_-maximum.

	PET/CT SUV_LBM_-maximum (2.13 ± 0.26)			PET/MRI SUV_LBM_-maximum (1.58 ± 0.29)			
*P *	0.001						
*R *	0.873						
*N *	10			10			

Model	Unstandardized coefficients		Standardized coefficients	*t *	*P *	95% confidence interval	
	*B *	Std. error	Beta			Lower	Upper

1 (constant)	−0.49	0.44		−1.11	0.301	−1.54	0.55
PET/CT SUV_LBM_-maximum	0.97	0.20	0.87	4.73	0.002	0.48	1.46

^a^Dependent variable: PET/MRI SUV_LBM_-maximum.

**Table 2:** Comparison of PET/CT SUV_BW_-maximum and PET/MRI SUV_BW_W-maximum.

	PET/CT SUV_BW_-maximum (2.85 ± 0.34)			PET/MRI SUV_BW_-maximum (2.14 ± 0.45)			
*P *	0.0001						
*R *	0.924						
*N *	10			10			

Model	Unstandardized coefficients		Standardized coefficients	*t *	*P *	95% confidence interval	
	*B *	Std. error	Beta			Lower	Upper

1 (constant)	−1.339	0.548		−2.445	0.044	−2.634	−0.044
PET/CT SUV_BW_- maximum	1.221	0.191	0.924	6.398	0.0001	0.770	1.672

^a^Dependent variable: PET/MRI SUV_BW_-maximum.

**Table 3:** Comparison of PET/CT SUV_BSA_-maximum and PET/MRI SUV_BSA_-maximum.

	PET/CT SUV_BSA_-maximum (0.78 ± 0.06)			PET/MRI SUV_BSA_-maximum (0.58 ± 0.07)			
*P *	0.026						
*R *	0.661						
*N *	10			10			

Model	Unstandardized coefficients		Standardized coefficients	*t *	*P *	95% confidence interval	
	*B *	Std. error	Beta			Lower	Upper

1 (constant)	−0.018	0.259		−0.068	0.948	−0.629	0.594
PET/CT SUV_BSA_- maximum	0.762	0.327	0.661	2.330	0.053	0.011	1.535

^a^Dependent variable: PET/MRI SUV_BSA_-maximum.

**Table 4:** Comparison of PET/CT SUV_LBM_-maximum and PET/MRI SUV_LBM_-maximum using a contrast media.

	PET/CT SUV_LBM_-maximum (2.08 ± 0.33)			PET/MRI SUV_LBM_-maximum (1.47 ± 0.27)			
*P *	0.001						
*R *	0.748						
*N *	16			16			

Model	Unstandardized coefficients		Standardized coefficients	*t *	*P *	95% confidence interval	
	*B *	Std. error	Beta			Lower	Upper

1 (constant)	0.19	0.33		0.58	0.572	−0.52	0.91
PET/CT SUV_LBM_-maximum	0.61	0.15	0.74	3.90	0.002	0.27	0.95

^a^Dependent variable: PET/MRI SUV_LBM_-maximum.

**Table 5:** Comparison of PET/CT SUV_BW_-maximum and PET/MRI SUV_BW_-maximum using a contrast media.

	PET/CT SUV_BW_-maximum (2.79 ± 0.48)			PET/MRI SUV_BW_-maximum (1.96 ± 0.33)			
*P *	0.001						
*R *	0.749						
*N *	16			16			

Model	Unstandardized coefficients		Standardized coefficients	*t *	*P *	95% confidence interval	
	*B *	Std. error	Beta			Lower	Upper

1 (constant)	0.545	0.368		1.482	0.164	−0.256	1.346
PET/CT SUV_BW_- maximum	0.508	0.130	0.749	3.916	0.002	0.225	0.790

^a^Dependent variable: PET/MRI SUV_BW_-maximum.

**Table 6:** Comparison of PET/CT SUV_BSA_-maximum and PET/MRI SUV_BSA_-maximum using a contrast media.

	PET/CT SUV_BSA_-maximum (0.77 ± 0.11)			PET/MRI SUV_BSA_-maximum (0.54 ± 0.10)			
*P *	0.022						
*R *	0.648						
*N *	16			16			

Model	Unstandardized coefficients		Standardized coefficients	*t *	*P *	95% confidence interval	
	*B *	Std. error	Beta			Lower	Upper

1 (constant)	−0.034	0.140		0.242	0.813	−0.272	0.340
PET/CT SUV_BSA_- maximum	0.658	0.658	0.728	3.682	0.023	0.569	1.048

^a^Dependent variable: PET/MRI SUV_BSA_-maximum.

**Table 7:** According to noncontrast media or contrast media used, SUV_LBM_-maximum normal volumes corresponding to PET/CT and PET/MRI.

	Paired differences				*R *	*P *	Red (%)	Paired differences				*R *	*P *	Red (%)
	Mean	Std. error	*N *	* t *				Mean	Std. error	*N *	*t *			
PET/CT liver	2.0 ± 0.3	0.10	10	10.7	0.860	0.0001	30.6	1.9 ± 0.2	0.06	16	9.7	0.622	0.0001	32.6
PET/MRI liver	1.4 ± 0.1	0.06	10					1.3 ± 0.2	0.07	16				
PET/CT spleen	1.5 ± 0.1	0.05	10	6.8	0.250	0.0001	32.1	1.5 ± 0.2	0.07	16	11.5	0.761	0.0001	35.8
PET/MRI spleen	1.1 ± 0.1	0.04	10					1.0 ± 0.2	0.06	16				
PET/CT aorta	1.3 ± 0.1	0.05	10	4.6	0.548	0.002	18.2	1.4 ± 0.2	0.05	16	3.7	−0.053	0.002	22.8
PET/MRI aorta	1.1 ± 0.1	0.06	10					1.0 ± 0.2	0.06	16				
PET/CT L-1	1.9 ± 0.4	0.15	10	3.2	0.658	0.012	21.1	1.6 ± 0.4	0.11	16	7.0	0.907	0.0001	23.6
PET/MRI L-1	1.5 ± 0.4	0.14	10					1.2 ± 0.5	0.13	16				
PET/CT L-2	1.9 ± 0.4	0.14	10	3.6	0.702	0.006	24.7	1.9 ± 1.2	0.33	16	3.1	0.897	0.007	27.3
PET/MRI L-2	1.4 ± 0.5	0.17	10					1.3 ± 0.8	0.22	16				
PET/CT L-3	1.8 ± 0.4	0.14	10	6.0	0.828	0.0001	27.6	1.6 ± 0.3	0.07	16	6.6	0.569	0.0001	30.6
PET/MRI L-3	1.3 ± 0.4	0.14	10					1.1 ± 0.3	0.08	16				
PET/CT L-5	0.7 ± 0.0	0.02	10	4.5	−0.371	0.002	30.5	0.6 ± 0.1	0.02	16	6.8	0.497	0.0001	30.7
PET/MRI L-5	0.5 ± 0.1	0.03	10					0.4 ± 0.1	0.02	16				
PET/CT cerebellum	5.7 ± 1.0	0.33	10	10.7	0.914	0.0001	25.8	5.9 ± 1.2	0.32	16	11.7	0.892	0.0001	31.3
PET/MRI cerebellum	4.2 ± 0.9	0.31	10					4.0 ± 0.8	0.22	16				

Total							26.3							29.3

**Table 8:** According to noncontrast media or contrast media used, SUV_BW_-maximum normal volumes corresponding to PET/CT and PET/MRI.

	Paired differences				*R *	*P *	Red (%)	Paired differences				*R *	*P *	Red (%)
	Mean	Std. error	*N *	* t *				Mean	Std. error	*N *	* t *			
PET/CT liver	2.6 ± 0.3	0.11	10	11.4	0.823	0.0001	30.4	2.6 ± 0.4	0.11	16	12.4	0.732	0.0001	35.8
PET/MRI liver	1.8 ± 0.1	0.05	10					1.6 ± 0.3	0.08	16				
PET/CT spleen	2.1 ± 0.1	0.04	10	6.7	0.338	0.0001	27.4	2.1 ± 0.3	0.10	16	10.0	0.717	0.0001	34.7
PET/MRI spleen	1.5 ± 0.2	0.08	10					1.3 ± 0.3	0.08	16				
PET/CT aorta	1.8 ± 0.2	0.08	10	3.7	0.594	0.006	15.7	1.8 ± 0.2	0.05	16	3.6	0.112	0.003	17.6
PET/MRI aorta	1.5 ± 0.2	0.07	10					1.4 ± 0.2	0.07	16				
PET/CT L-1	2.5 ± 0.5	0.18	10	2.7	0.656	0.023	19.3	2.1 ± 0.7	0.19	16	3.4	0.743	0.004	21.0
PET/MRI L-1	2.0 ± 0.7	0.23	10					1.7 ± 0.6	0.17	16				
PET/CT L-2	2.5 ± 0.5	0.18	10	3.5	0.749	0.007	23.3	2.6 ± 1.9	0.51	16	2.9	0.919	0.011	28.3
PET/MRI L-2	1.9 ± 0.7	0.24	10					1.9 ± 1.2	0.33	16				
PET /CT L-3	2.4 ± 0.5	0.19	10	5.3	0.825	0.001	27.2	2.1 ± 0.4	0.12	16	5.8	0.488	0.0001	32.5
PET/MRI L-3	1.7 ± 0.6	0.21	10					1.4 ± 0.4	0.11	16				
PET/CT L-5	0.9 ± 0.1	0.04	10	4.5	−0.149	0.002	31.9	0.8 ± 0.1	0.03	16	5.5	0.178	0.0001	29.4
PET/MRI L-5	0.6 ± 0.1	0.03	10					0.6 ± 0.1	0.03	16				
PET/CT cerebellum	7.6 ± 1.4	0.47	10	10.5	0.929	0.000	25.1	7.9 ± 1.5	0.40	16	11.8	0.871	0.0001	31.3
PET/MRI cerebellum	5.7 ± 1.4	0.48	10					5.4 ± 1.0	0.27	16				

Total							25.0							28.8

**Table 9:** According to noncontrast media or contrast media used, SUV_BSA_-maximum normal volumes corresponding to PET/CT and PET/MRI.

	Paired differences				*R *	*P *	Red (%)	Paired differences				*R *	*P *	Red (%)
	Mean	Std. error	*N *	* t *				Mean	Std. error	*N *	*t *			
PET/CT liver	0.7 ± 0.0	0.02	10	12.3	0.764	0.0001	31.0	0.7 ± 0.0	0.02	16	14.6	0.697	0.0001	36.1
PET/MRI liver	0.5 ± 0.0	0.01	10					0.4 ± 0.0	0.02	16				
PET/CT spleen	0.5 ± 0.0	0.01	10	6.6	-0.490	0.0001	27.5	0.5 ± 0.1	0.03	16	9.9	0.781	0.0001	36.2
PET/MRI spleen	0.4 ± 0.0	0.01	10					0.3 ± 0.1	0.02	16				
PET/CT aorta	0.5 ± 0.0	0.02	10	4.6	0.582	0.002	18.0	0.5 ± 0.0	0.01	16	3.2	0.363	0.007	18.0
PET/MRI aorta	0.4 ± 0.0	0.01	10					0.4 ± 0.1	0.02	16				
PET/CT L-1	0.7 ± 0.1	0.04	10	3.0	0.451	0.015	21.1	0.6 ± 0.1	0.05	16	6.8	0.924	0.0001	25.0
PET/MRI L-1	0.5 ± 0.1	0.04	10					0.4 ± 0.2	0.05	16				
PET/CT L-2	0.7 ± 0.1	0.04	10	3.6	0.540	0.006	25.7	0.7 ± 0.4	0.13	16	2.9	0.888	0.011	26.3
PET/MRI L-2	0.5 ± 0.1	0.05	10					0.5 ± 0.3	0.08	16				
PET/CT L-3	0.6 ± 0.1	0.04	10	5.6	0.725	0.001	28.7	0.6 ± 0.1	0.03	16	6.1	0.560	0.0001	31.6
PET/MRI L-3	0.4 ± 0.1	0.04	10					0.4 ± 0.1	0.03	16				
PET/CT L-5	0.2 ± 0.0	0.01	10	4.8	0.175	0.001	33.3	0.2 ± 0.0	0.01	16	5.7	0.423	0.0001	30.4
PET/MRI L-5	0.1 ± 0.0	0.01	10					0.1 ± 0.0	0.01	16				
PET/CT cerebellum	2.1 ± 0.3	0.10	10	9.5	0.848	0.0001	26.1	2.1 ± 0.3	0.08	16	12.8	0.799	0.0001	31.5
PET/MRI cerebellum	1.5 ± 0.2	0.09	10					1.5 ± 0.2	0.06	16				

Total							26.4							28.2

## Appendices

### A. Resource of PET/MRI Used for Examination

The acquisition variables of MRI image are as follows: integrated parallel acquisition technique factor 2, voxel size 4.1 × 2.6 × 3.1 mm^3^ (in-plane resolution × slice thickness), acquisition time 18 s, repetition time (TR)/echo time (TE) 3.6/1.225, matrix 79 × 192, number of excitations 1, FOV 500 mm, phase FOV 72%, 1 slab with 128 slices, slice thickness 2.6 mm, flip angle 10°, and bandwidth 960 kHz. This is introduced in Yeungnam University Hospital, Republic of Korea, on July 16, 2012 (Figure 2).

### B. Resource of PET/CT Used for Examination

Figure 3.
